# Lived experience of people with cryptococcal meningitis: A qualitative study

**DOI:** 10.4102/sajhivmed.v25i1.1560

**Published:** 2024-05-29

**Authors:** Neo A. Legare, Vanessa C. Quan, Nelesh P. Govender, Jane W. Muchiri

**Affiliations:** 1 School of Health Systems and Public Health, Faculty of Health Sciences, University of Pretoria, Pretoria, South Africa; 2 Division of Public Health Surveillance and Response, GERMS-SA, National Institute for Communicable Diseases, Division of the National Health Laboratory Service, Johannesburg, South Africa; 3 Centre for Healthcare-Associated Infections, Antimicrobial Resistance and Mycoses, National Institute for Communicable Diseases, Division of the National Health Laboratory Service, Johannesburg, South Africa; 4 Clinical Microbiology and Infectious Diseases, Faculty of Health Sciences, University of the Witwatersrand, Johannesburg, South Africa; 5 Department of Human Nutrition, Faculty of Health Sciences, University of Pretoria, Pretoria, South Africa

**Keywords:** HIV-associated cryptococcal meningitis, lived experiences, qualitative study, Johannesburg, routine care

## Abstract

**Background:**

The high burden of cryptococcal meningitis (CM) among people living with HIV persists despite widespread access to antiretroviral therapy. Efforts to prevent CM among people living with HIV could be hindered by a limited understanding of their lived experiences of CM and its diagnosis.

**Objectives:**

To explore and describe the experiences of people diagnosed with HIV-associated CM in routine care. Two public healthcare facilities in Johannesburg, South Africa.

**Method:**

This was a qualitative-methods exploratory, descriptive, phenomenological study. We conducted semi-structured, individual in-depth interviews with nine purposively sampled participants (comprising 5 men and 4 women). Data were analysed using the Moustakas phenomenological approach.

**Results:**

Five themes and several sub-themes emerged from the data. Participants described their experiences of being diagnosed, which were marked by intense headaches. Diagnosis of CM led to reduced quality of life, fear of death, and loss of income. Participants described their CM treatment experience and health-seeking behaviour including self-medication, seeking help from traditional healers and general practitioners and utilising public health facilities as a last resort. Barriers to care included negative healthcare workers’ attitudes, unhealthy lifestyles, and poor knowledge of CM.

**Conclusion:**

People with HIV-associated CM face negative impacts prior to and after diagnosis. These patients struggled to access timely quality healthcare. Patients starting or restarting antiretroviral therapy, and thus at risk for CM, should receive CM education as part of HIV counselling.

**What this study adds:** Our findings highlight people’s lived experiences with HIV-associated cryptococcal meningitis (CM) in routine care. People with HIV-associated CM face personal and health-system hurdles that hinder them from receiving timely access to quality care.

## Introduction

Globally, approximately 152 000 incident cases of HIV-associated cryptococcal meningitis (CM) occur each year, accounting for 19% of AIDS-related deaths.^1^ Africa shoulders a majority (54%) of these incident cases and South Africa has the highest estimated burden (i.e., 23 000 cases per annum).^1^ A retrospective study reported that CM accounted for 62% (*n* = 7406) of microbiologically confirmed cases of meningitis between 2009 and 2012 in Gauteng, South Africa.^2^ A surveillance programme in South Africa (GERMS-SA) reported that the national incidence of laboratory-confirmed CM in 2021 was 67 cases per 100 000 people living with HIV.^3^ The incidence of CM in South Africa has decreased since the peak approximately a decade ago.^3^ This might be associated with people starting antiretroviral therapy (ART) at an earlier stage of their HIV infection or by prevention of meningitis through the cryptococcal antigenaemia (CrAg) screen-and-treat programme.^3^ Most CM cases now occur among people who have interrupted ART.^3,4^ Notably, most local studies only report laboratory-confirmed cases of CM in South African public healthcare facilities, and the actual disease burden may be underestimated, with many patients dying at home without seeking care.^2,5,6,7^

People with advanced HIV disease (defined as a CD4+ T-cell count below 200 cells/µL) have an elevated risk of CM.^8,9^ The two strategies for CM prevention and treatment, namely CrAg screening and shorter-course induction antifungal therapy, have lowered mortality in this population.^10,11^ A randomised-controlled trial in Tanzania and Zambia showed that early detection through CrAg screening coupled with ART adherence support prevented CM and lowered mortality rates.^12^ In South Africa, a CrAg screening programme was implemented in 2016.^13^

Treatment of CM lasts for at least 12 months and comprises induction, consolidation, and maintenance phases. In the induction phase, the fungus is rapidly cleared from the body, consolidation mops up fungi not completely eradicated, and maintenance aims to prevent CM from recurring by suppressing fungal growth.^8,11^ The World Health Organization (WHO) strongly recommends using single-dose liposomal amphotericin B followed by 5-flucytosine (5-FC), and fluconazole for 2 weeks as the preferred induction regimen.^8,14^ Evidence from African clinical trials supports the WHO recommendations of optimised short-course antifungal combinations of amphotericin B, 5-FC, and fluconazole.^8,15^ Flucytosine is currently on the South African Essential Medicines List following its registration with the South African Health Products Regulatory Authority in 2021; 5-FC is included in a national tender.

Currently, CM in people living with HIV is reasonably well understood in terms of genetic predisposition to infection, protective immune responses, immune-mediated pathology, and epidemiology.^7,16^ Despite CM being the second leading cause of mortality after tuberculosis (TB) in people living with HIV, most studies focus on the lived experiences of people living with either HIV or TB. The few qualitative-methods studies on CM, exploring aspects such as acceptability of different CM regimens, have been done within the context of clinical trials.^17,18,19,20^ Thus there is a gap in understanding the lived experience of people with CM outside clinical trials. Hence, we explored the experiences of people living with HIV-associated CM in routine care using qualitative methods.

## Research methods and design

### Study design

This was a qualitative-methods exploratory, descriptive, and phenomenological study. Phenomenological studies aim to describe shared meaning derived from the lived experiences of several individuals.^21^

### Setting

The study was conducted at the Chris Hani Baragwanath Academic Hospital (CHBAH) and Hillbrow Community Health Centre (CHC), which are public health facilities in Gauteng, South Africa. The CHBAH serves an immediate population of at least 1.5 million people living in Soweto and its surroundings while also serving as the tertiary referral centre for much of the Gauteng population (11.4m).^22^ Hillbrow CHC is located in the centre of the Johannesburg Central Business District.^22^ The CHC provides primary healthcare services to the local community including HIV testing and treatment. People who live within the catchment area of the Hillbrow CHC are primarily immigrants and migrant workers who have come to the city for work purposes.^23^ The CHBAH is an enhanced surveillance site within the GERMS-SA laboratory-based surveillance network in South Africa.

### Study population and sampling strategy

The target population included people living with HIV (outpatients and inpatients), who were older than 18 years with laboratory-confirmed CM. Participants had to have a Glasgow Coma Scale score of 15 and living with a diagnosis of CM for more than 10 days and 30 days for inpatients and outpatients, respectively. We purposively selected 9 people living with HIV who met these inclusion criteria. Phenomenological studies typically only require data from a few individuals who have experienced the phenomenon to discover its core elements.^24^

For inpatients, we screened the GERMS-SA database to identify prospective participants. Eligible patients were asked to participate, and their consent was sought. Outpatients were identified through pharmacy records; at Hillbrow CHC we retrieved the medical records of patients on fluconazole to assess their eligibility. Eligible patients were contacted by phone to confirm the date of their next clinic appointment. At this clinic visit, their consent to participate was sought.

### Data collection

Data were collected using a semi-structured interview guide during individual face-to-face, in-depth interviews. Interviews were conducted from September 2020 until November 2021. All interviews were audio-recorded, and field notes were taken. To maintain privacy, patients were interviewed in their cubicles and curtains were closed. Outpatients were interviewed in the consultation room. During the interviews, we adhered to coronavirus disease 2019 (COVID-19) safety protocols including face masking and maintaining a distance of 1 m. Interviews were conducted in the participants’ preferred language by the first investigator, who is conversant with the main local languages (Sepedi, IsiZulu, Sesotho and Setswana) spoken in the setting. Interviews lasted for 35 min to 60 min. The interview started by asking a broader general question, ‘*What has it been like living with CM*?’. Other open-ended questions were asked to provide a textural and organisational description of the experience of living with CM.

### Data analysis

The interview recordings were transcribed verbatim by an independent provider. Interviews conducted in the participants’ home languages were translated into English by the researcher, and then transcribed.

Data were analysed manually by the researcher using the Moustakas phenomenological approach, which is a modification of the Stevick-Colaizzi-Keen method.^25^ The four steps of the Moustakas approach are as follows:

1. Horizontalisation of data: the transcripts and field notes were read repeatedly to identify statements about how participants experienced CM. The statements were recorded in a Microsoft Excel spreadsheet to develop a list of statements without overlapping or repeating information.
2. Individual textural descriptions: related statements were grouped into units or sub-themes.
3. Composite structural description: sub-themes were further studied in terms of the objectives to create or identify different inventive and creative ways for possible meaning and different viewpoints about how the participants experienced CM.
4. Textural structure: the sub-themes were further interrogated and related sub-themes combined to formulate final themes.

Trustworthiness was maintained by upholding the principle of credibility, transferability, conformability, and dependability.^21^ Credibility was maintained by the extended period of the study.^21^ The researcher also read the transcripts while listening to the audio for quality checks. Transferability was ensured by describing the sampling methods and findings in detail so that they were transparent.^21^ Conformability was ensured by keeping audits trails of all actions via a Microsoft Word document as a means of opening the research process to outside inspection.^21^ Additionally, each step of the analysis was discussed with the co-authors, one of whom is experienced in qualitative-methods research. Further, the research process and findings were discussed with colleagues who are experts in the field as part of peer debriefing. Dependability was ensured by concurrently collecting and analysing data, which helped to refine some of the interview questions.^21^

### Ethical considerations

Ethical approval was obtained from the Faculty of Health Science Research Ethics Committee of the University of Pretoria, reference no. 433/2020. Permission to conduct the study at the CHBAH was obtained from the Medical Advisory Committee and Hospital Chief Executive Officer. Permission from the Hillbrow CHC was sought from the Research Committee of the Johannesburg Health District, reference number 2020_06_005. Written informed consent was obtained before conducting interviews. With participants’ permission, interviews were audiotaped. Participants’ names were not used during interviews to maintain confidentiality during transcribing.

## Results

### Participant characteristics

A total of 9 patients (5 men, 4 women) aged between 22 years and 47 years, and who were mainly black people (*n* = 8), participated in the study. One participant was mixed race.

### Participants’ experiences of cryptococcal meningitis

Five themes and several sub-themes were identified (Table 1).

**TABLE 1:** Themes and sub-themes emerging from the lived experiences of people with HIV-associated cryptococcal meningitis.

Major themes	Sub-themes
Experience of CM symptoms	Signs and symptoms and their severity
Impact of CM diagnosis and symptoms	Quality of life
	Fear of dying
	Loss of income
CM treatment experience	Therapeutic lumbar puncture
	Effectiveness of treatment
Health-seeking behaviour for symptoms	Self-medicating
	Traditional/spiritual healer, general practitioner consultation
Barriers to HIV and CM care	Negative healthcare worker attitude
	Unhealthy lifestyle
	Poor knowledge of CM

CM, cryptococcal meningitis.

### Experience of cryptococcal meningitis symptoms

All participants visited healthcare facilities because they had severe headaches for more than 2 weeks. Other signs included painful neck or neck stiffness, vomiting, and painful eyes when looking into the light. Most participants had headaches that were persistent throughout the day and could not recall the exact time and place when the pain started. Participants initially thought it was just a headache that would go away, but they realised that the headache was not getting any better, and they had to seek help. Headaches did not start in any specific area, with some participants indicating the frontal part of the head and others from the back and the neck upwards. Participants explained that the pain could not be resolved at home and that the pain was only relieved once they were admitted to the hospital:

‘I just stand up then I had this huge headache. It looks like my head. I can’t describe to you. Like it’s going to tear out on here.’ (Participant 2, Female, 38 years old)

Participants described the pain as being more severe than any other pain they had ever experienced, even worse than labour pains:

‘The pain is more severe as compared to the labour pain. I have three children, but I never had a big pain that I got here from that. It’s better [*labour pains*]. You forget about it, but I’ll never forget about that pain.’ (Participant 1, Female, 37 years old)

‘No, nothing. Nothing. Even to labour can’t even compare, things like. The labour pains are much better than this pain.’ (Participant 2, Female, 38 years old)

One male patient who had been shot with a gun in his leg indicated that even the gunshot pain was much better than the pain he felt due to CM: ‘even a gunshot wound is less painful than this’ (Participant 5, Male, 22 years old).

### Impact of cryptococcal meningitis diagnosis and symptoms

Participants acknowledged that their lives changed once they developed CM symptoms. Some participants reported a loss of income due to loss of jobs while others reported being afraid of death. Additionally, participants perceived a reduced quality of life. Most participants were unemployed, but some had temporary jobs. Some of those with temporary jobs had to stop working to stay at home, spending their time in bed sleeping, and resting to relieve the pain, ‘I lost my job so really from here finish this … medication course, start the ARVs, get a job’ (Participant 5, Male, 22 years old).

Participants reported a poorer quality of life and an increased sense of being more dependent on others. Participants felt as if the pain had changed them and felt as if they were no longer themselves due to the changes affecting their daily routines and lifestyles. Some participants felt that they were a burden to their families because they could not perform certain tasks:

‘It makes me feel bad. It makes, I don’t know, I don’t even know. I can’t even explain how it makes me feel. I don’t like it.’ (Participant 2, Female, 38 years old)

Some participants reported feeling depressed about their condition. Participants isolated themselves from friends to avoid being asked about their health, especially if they had notable weight loss:

‘Nah it makes me feel depressed because whenever someone … if my friends see me, where have you been? I’m like, I’ve been around. What’s wrong? Nothing’s wrong. But obviously, they could see the weight was going down.’ (Participant 5, Male, 22 years old)

Participants were afraid of succumbing to CM and its sequelae. Some of them mentioned they had heard about meningitis and someone who had died from it, although they did not know the type of meningitis. Others knew of family members or other people who had meningitis but never recovered fully and were still struggling with hearing loss, loss of vision, weakness of limbs, and cognitive slowness:

‘I didn’t even know, how do they know, only my mother says, my mother is a nurse, and only she said that in her younger days the people died of meningitis because there wasn’t a cure for it. All the babies because my cousin is blind. Ja, and he got meningitis.’ (Participant 1, Female, 37 years old)

‘First thing I saw that it’s deadly, brain-damaging and what else? Yeah, I think those are the two that sparked me. Brain damage.’ (Participant 5, Male, 22 years old)

### Cryptococcal meningitis treatment experience

Regarding treatment, participants described their experiences with therapeutic lumbar puncture (LP) and their perceptions on the effectiveness of treatment. Most participants with serial LPs to decrease intracranial pressure mentioned that LPs helped to relieve headaches. Three participants noted that the LP procedure was painful and did not help; these 3 participants only had an LP at diagnosis. In contrast, 6 participants reported feeling better every time the doctor did an LP:

‘An Indian doctor and another doctor did it. They did it so nicely. Instantly when it just was finished here with my back, then all the pain went up, everything went away.’ (Participant 1, Female, 37 years old)

All participants indicated that they received better care at the hospital than that offered by general practitioners (GPs) before being admitted to the hospital. Inpatients felt they received effective treatment from primary healthcare clinics (PHCs) and the hospitals. Participants were referred from PHCs to the hospital. Few participants were aware of the medication they received in hospital. However, they acknowledged that they felt the medication was effective:

‘The pill, the drip helped me. They call it a mellow yellow drip, yes. It’s yellow. It helped me a lot. So I had to take fourteen litres for fourteen days. It’s for two weeks. I completed a course yesterday.’ (Participant 1, Female, 37 years old)

### Health-seeking behaviour for symptoms

**FIGURE 1:**
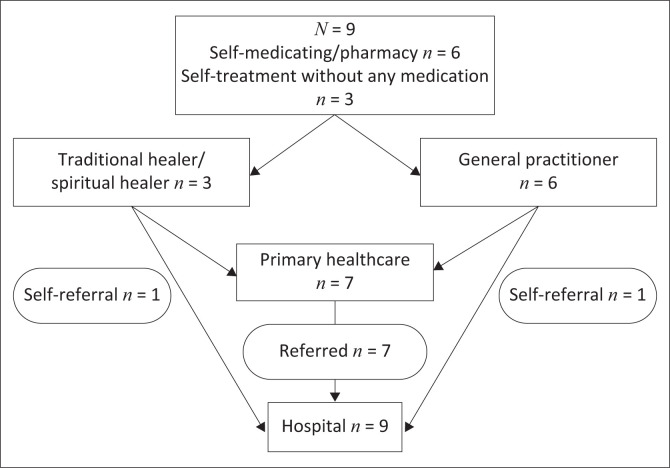
Summary of pathways taken by people seeking treatment after cryptococcal meningitis signs and symptoms.

Health-seeking behaviour emerged as a strong theme. Participants initially sought help for their symptoms from other sources and not from health professionals. Six out of 9 participants self-medicated with over-the-counter analgesics such as paracetamol, aspirin, or combinations of these, while 3 participants were self-treated without medication. The spiritual or traditional healers and GP were seen as the next option after self-medication or self-treatment failed to improve their symptoms (Figure 1). The 3 participants who did not use any medication went to the traditional healers while the 6 participants who used over-the-counter medications consulted the GP (Figure 1):

‘I just got some tablets, Panado^®^ and Disprin^®^, just that.’ (Participant 7, Male, 45 years old)

‘Just buying the over-the-counter medication just thinking it’s just a headache, and then after I went to the GP, then still no change. I went to the GP again, they say that it’s migraine, but then still no change. That is when I decided to go to the clinic.’ (Participant 2, Female, 38 years old)

‘I went to the prophet. Who also said the same thing that I am saying, that they are muthi-ing [*bewitching*] me. But didn’t give me any medication or something, he was just prophesising.’ (Participant 3, Male, 44 years old)

Participants only sought help from PHC facilities (*n* = 7) or self-referred directly to the tertiary hospital (*n* = 2) when their headaches were unresolved. Seven participants who went to the local clinic were referred to the hospital at first contact, while the remaining participant self-referred to the hospital:

‘When I go to the clinic, they just gave me the transfer to the hospital.’ (Participant 2, Female, 38 years old)

Notably, participants who consulted GPs were never referred to the next level of care despite their symptoms not improving.

### Barriers to HIV and cryptococcal meningitis care

Participants identified healthcare workers’ negative attitudes, their unhealthy lifestyles, and poor CM knowledge as barriers to effective HIV and CM care.

Some participants mentioned that they received terrible treatment from their local clinics. Participants felt that healthcare workers had negative attitudes, discouraging them from collecting their medication every month, especially for employed participants who could not take sick leave from work each month. One participant indicated that she had to send her child to collect her treatment as she could not take leave every month, but the nurse would not allow that:

‘At the clinic, they are so rude. You see me. I’m a domestic worker. I clean for the pastor. I clean every day. Now I send my children to go and fetch the pills. Then they don’t want to give those children the pills. Then they say I must come. Why must I come? And when they, I need that money, you see? I can’t say to the pastor that I can’t work today. I must go to the clinic. The pastor doesn’t want to hear that.’ (Participant 1, Female, 37 years old)

Stigma emerged as a sub-theme. One participant indicated that he used to collect his treatment in the private sector using medical aid. He lost his job and income, and his medical aid was cancelled. He felt that he could not collect his medication at a public clinic because healthcare workers had negative attitudes toward people who are HIV-positive:

‘I was on medical aid. Then after that time that stigma, that local clinics, sisters there, they like to make noise, they wanna shout at me that you’re still young and whatever you know yeah.’ (Participant 5, Male, 22 years old)

Participants also reported using alcohol, which caused them to default on ART. One participant indicated that he was consuming a lot of alcohol, especially during the festive season. Consequently, he forgot to take his medication because he was drunk by the time he had to take his medicine:

‘I think because last November I didn’t drink my medication, I think, November and December. I think maybe, it is the cause, I don’t know. Every day I was drinking and the timings I had to drink pills, I was drunk.’ (Participant 6, Male, 41 years old)

Poor CM knowledge stood out as a factor contributing to participants visiting unconventional places for treatment, including traditional healers. Participants’ understanding of CM was poor because some linked the symptoms with being bewitched, stressed, or being hit on the head. None of the participants linked CM to HIV or ART non-adherence. Some participants viewed the headaches as a spiritual attack or some form of witchcraft, and therefore they had to seek spiritual help from traditional healers. Some participants related that when they approached traditional healers, they were informed that someone was behind their headaches. Furthermore, the fact that the headache was persistent and severe was reason for some participants who did not believe in witchcraft to believe they had been bewitched:

‘When it started, no, but along the way, I thought that maybe someone is muthi-ing me, yes. I went to the prophet. He said I should pray, who also said the same thing that I am saying, that they are muthi-ing me. But they didn’t give me any medication or something. He was just prophesising.’ (Participants 3, Male, 44 years old)

‘So, it was so stressful. So stressful, because I owe people who borrowed me money for transport. I had to pay them back and they don’t pay us, so the headache started.’ (Participant 2, Female, 38 years old)

‘I was smoking drugs with my child’s father. I used to fall hard on my head.’ (Participant 1, Female, 37 years old)

‘I have it every day when I’m at that house. When something goes wrong in my house, I have it every day and I’m not wrong. I don’t believe in it, but the things that happen make you find, make you believe in it [*witchcraft*].’ (Participant 1, Female, 37 years old)

## Discussion

This study investigated the lived experiences of people with HIV-associated CM in routine care who reported negative experiences related to symptoms and the impact on their lives, including income loss, diminished quality of life, depression, and fear of dying. Additionally, negative experiences were associated with delays in receiving appropriate care, which stemmed from late hospital presentation and misdiagnosis by GPs and traditional healers. Limited understanding of CM appeared to influence participants’ health-seeking behaviour such as self-medicating and consulting traditional healers. Healthcare workers’ negative attitudes and participants’ unhealthy lifestyles were the main barriers to both HIV and CM care during the continuation phase, despite participants having positive experiences of CM treatment at the hospital.

Some of the findings from our study corroborate those of a qualitative-methods study on pathways to care conducted in Botswana and Uganda among participants with HIV-associated CM by Lawrence et al.^26^ Similar to our findings, that study found that delays in CM diagnosis and treatment contributed to the length of the pathway to care.^26^ Headaches were a common complaint, usually with no serious repercussions, and frequently misdiagnosed by medical professionals as migraines rather than CM.^26^ Meningitis was rarely suspected because, in both investigations, people living with HIV were unaware that headaches could indicate a serious underlying illness in the context of advanced HIV disease.^26^

Headaches emerged as the most common symptom experienced by participants in our study, as also reported in the Ugandan and Botswana studies.^26^ In addition, clinical studies assessing CM presentation have reported headaches as a common symptom in between 93% and 100% of cases.^26,27,28^ Our findings suggest that these headaches were more severe than other symptoms.

Our findings highlight the negative impact of CM on patients’ lives, including low quality of life, income loss, and fear of dying. Most participants lacked permanent jobs and lost their source of income due to hospitalisation, reflecting a common issue among people living with HIV who often miss clinic visits due to work commitments. In Russia, people living with HIV were motivated to remain on ART because they wanted to avoid poor quality of life and were afraid of dying.^29^ In contrast, participants in our study cited these factors as barriers to ART adherence, which may have increased their risk for CM. People living with HIV in other settings are also afraid of loss of income and fear of dying, but it seems that stigma, barriers to care, and not having enough information contribute to treatment non-adherence.^30^

In resource-limited settings, a single-dose liposomal amphotericin B with 14 days of 5-FC and fluconazole is an effective induction therapy for CM.^15,31^ Participants in this study generally tolerated CM treatment in hospital but after discharge, many CM patients are lost to follow-up.^32^ This trend was evident when enrolling patients from the outpatient department at Hillbrow CHC where most CM patients did not return for follow-up. In South Africa, therapeutic LPs are part of CM treatment for patients with raised intracranial pressure.^9,33,34^ Participants who underwent therapeutic LPs reported improved pain control and less pain than when LPs were performed only for diagnosis.^8,9^

Our findings emphasise the important role of staff attitudes at local clinics as a primary barrier to ART non-adherence or attending clinics to receive more ART. Similar findings have been reported in a study investigating extensively drug-resistant TB, where patients reported discrimination and being humiliated by nurses when collecting medication. These patients described primary healthcare staff as being unsupportive and unhelpful when seeking medical assistance.^35^

Poor knowledge about CM, as evidenced by participants not associating the headaches with HIV and consulting traditional or spiritual healers, was identified as another barrier to care in this study. These results agree with the Uganda and Botswana study that also found poor knowledge of CM as a barrier to care.^26^ In that study, a majority of participants had never heard of meningitis; hence they did not associate HIV with CM.^26^ Some participants also consulted traditional healers. Poor knowledge of CM thus is a contributor to diagnosis delay. Similar studies on TB and HIV suggest that patients’ belief systems, including consultations with traditional healers, can delay TB treatment.^36^ A multi-country qualitative-methods study highlighted how patients living with HIV often switch between biomedical, traditional, and faith-based healthcare systems, causing obstacles in the HIV care cascade if they prioritise alternatives over complementary care.^37^ A study in KwaZulu-Natal found that 40% of patients with TB had visited traditional healers before being diagnosed with TB.^38^

A study on perceived barriers to HIV care in Kenya found that many people sought care at public healthcare facilities.^30^ In contrast, participants in our study initially used over-the-counter medications or self-medicated to alleviate headaches and eventually 3 participants turned to traditional or spiritual healers when the pain persisted. Only after these alternative measures failed, did participants seek help from the healthcare system, either through PHCs or GPs. These results agree with the study by Lawrence et al.,^17^ which also indicated participants resorted to using analgesics or herbal remedies while others consulted traditional healers before seeking healthcare from health facilities. A quantitative-methods study exploring the care pathways for CM patients in South Africa reported the pre-diagnosis health-seeking behaviour of CM patients and found that most went to PHCs (50%), followed by hospitals (22%), GPs (14%), pharmacies (8%), and traditional healers (2%).^32^

### Strength and limitations

This qualitative-methods study is the first in South Africa to explore HIV-associated CM experiences in routine care, filling an important knowledge gap. Recruitment of participants posed a significant challenge related to inability to trace outpatients on maintenance fluconazole at the PHC and the severity of CM in hospitalised patients. The use of a sample from one tertiary facility and one PHC setting also requires caution in generalising the results to other populations or settings.

### Implications or recommendations

CM-related health education should be integrated into post-HIV counselling for patients starting ART who are at risk of CM (e.g., CD4 count ≤ 200 (cells/µL), positive reflex CrAg test, and high HIV viral loads). Patients who are aware of the symptoms may be more likely to seek early treatment for CM. Further studies are needed to understand healthcare workers’ attitudes and assess the knowledge of GPs on the current guidelines for the diagnosis, prevention, and management of cryptococcal disease in adults.

## Conclusion

This study reveals that patients with HIV-associated CM face adverse consequences prior to and upon diagnosis, alongside personal and health-system challenges that impede timely access to quality healthcare. These challenges contribute to delayed diagnosis and linkage to care, resulting in late presentation. Barriers identified in this study may explain poor retention in care and virological failure on ART among patients with HIV-associated CM.
